# Mental Health Outcomes Among International College Students in the COVID-19 Pandemic: Protocol for a Scoping Review

**DOI:** 10.2196/91839

**Published:** 2026-09-25

**Authors:** Kruti S Chaliawala, George A Kelley

**Affiliations:** 1 School of Public and Population Health, Boise State University, 1910 University Drive, Boise, ID, 83725, United States, 1 (208) 426-3921

**Keywords:** anxiety, COVID-19, depression, international college students, mental health, scoping review, stress, suicidal ideation

## Abstract

**Background:**

International college students in the United States experience unique academic, cultural, financial, and immigration-related challenges that place them at increased risk of adverse mental health outcomes, including stress, anxiety, depression, and suicidal ideation. The COVID-19 pandemic further intensified these challenges through disruptions in education, travel restrictions, social isolation, and uncertainty regarding immigration and visa policies. Although numerous studies have examined international students’ mental health during this period, the available evidence has not been comprehensively mapped to characterize the extent, nature, and methodological characteristics of the literature or to identify gaps requiring further investigation.

**Objective:**

The objective of this scoping review is to systematically map the available evidence on stress, anxiety, depression, and suicidal ideation among international college students enrolled in US institutions during and following the COVID-19 pandemic, describe the characteristics of the existing literature, and identify knowledge gaps to inform future research, policy, and practice.

**Methods:**

This protocol follows the Joanna Briggs Institute methodology for scoping reviews and the methodological frameworks. The completed review will be reported in accordance with the PRISMA-ScR, and the search strategy will be developed and reported following the PRISMA-S recommendations. Electronic searches will be conducted in PubMed (MEDLINE), PsycINFO, CINAHL Complete, Scopus, ERIC, and Web of Science Core Collection for peer-reviewed English-language studies published between January 1, 2020, and December 31, 2025. Eligible studies will include quantitative, qualitative, and mixed methods research examining stress, anxiety, depression, or suicidal ideation among international college students enrolled in US institutions. The 2 independent reviewers will screen studies, extract data on study characteristics, participant demographics, mental health outcomes, measurement instruments, COVID-19-related contextual factors, and key findings, and synthesize the evidence using descriptive and narrative approaches. Consistent with scoping review methodology, no formal risk of bias assessment will be conducted.

**Results:**

The a priori protocol for this unfunded scoping review was registered with the Open Science Framework. We conducted the review from December 25, 2025, through April 17, 2026. After protocol registration, we added 2 additional reviewers to assist with study screening and data abstraction. After screening, 253 records underwent further eligibility assessment, and 16 studies met the eligibility criteria for inclusion. Data analysis and synthesis are complete, and the findings and discussion are being revised and formatted for manuscript submission. Publication of the completed review is tentatively anticipated by December 31, 2026.

**Conclusions:**

This scoping review will provide the first comprehensive evidence map of stress, anxiety, depression, and suicidal ideation among international college students enrolled in US institutions during and following the COVID-19 pandemic. The completed review will identify research gaps, summarize methodological trends, and provide evidence to inform future investigations, institutional initiatives, and policies aimed at improving mental health support for international college students.

## Introduction

### Rationale for the Scoping Review

University study is a period of psychological vulnerability for many individuals, especially international students, due to the challenges of relocation and acculturation [1,2]. International students contribute substantially to the academic, cultural, and economic vitality of US institutions of higher education; however, they frequently encounter barriers that place them at increased risk for adverse mental health outcomes compared with their domestic peers [3-6]. For example, while a previous systematic review and meta-analysis that included 35 studies representing 238,412 college students found comparable effects on mental health outcomes between domestic and international students, this study was not solely focused on international students in the United States [1]. In contrast, another systematic review of 64 studies focused on international undergraduate and graduate students in the United States found that psychological symptoms, such as stress, were statistically significant predictors of psychosocial adjustment [2].

International students in the United States experience heightened mental health challenges, exacerbated by the unique stressors of cultural adjustment, academic pressure, isolation, and reduced access to mental health resources specific to their needs [3,4,7,8]. These stressors frequently occur simultaneously and may reinforce one another, resulting in cumulative psychological burden and poorer academic, social, and health outcomes. In addition to traditional acculturative stressors, international students must often navigate complex immigration regulations, financial insecurity, employment restrictions, and uncertainty regarding visa policies, all of which may contribute to chronic stress and psychological distress [5,6,9,10]. Recent studies continue to demonstrate that international students report greater concerns regarding financial security, visa restrictions, employment uncertainty, and access to mental health services compared with many domestic students, suggesting that the effects of the pandemic have persisted beyond the initial public health emergency [5,6,9,10]. Furthermore, international students often underutilize counseling services because of stigma surrounding mental illness, cultural beliefs about help-seeking, concerns regarding confidentiality, and limited awareness of available campus resources [11-14]. Mental health stigma has also been identified as an important barrier to help-seeking during and after the COVID-19 pandemic, further limiting use of available support services among international students [8].

Cultural integration plays a critical role in the well-being of international students, as successful social and cultural integration can improve their sense of belonging and reduce feelings of loneliness and alienation [15]. Poor social connectedness has consistently been associated with elevated levels of stress, anxiety, and depressive symptoms among international students, emphasizing the importance of campus environments that promote inclusion and belonging [2,16-19]. However, many international students face barriers to integration with American students, further compounding their mental health struggles. The COVID-19 pandemic further intensified these vulnerabilities, with studies reporting higher rates of depression, anxiety, and other mental health problems among international students compared to domestic students [20,21]. For example, one study found that 18.8% of international students experienced moderately severe to severe depression, and 20.6% experienced severe anxiety during the pandemic [22]. Despite some improvement in mental health as the pandemic progressed, significant issues persisted [23,24], with ongoing concerns related to financial hardship, immigration uncertainty, discrimination, and access to culturally responsive mental health services [5,6,9,10]. Unfortunately, as the height of the COVID-19 period waned, international students continued to face enduring mental health challenges [20]. Recent evidence suggests that these challenges have extended into the postpandemic period, highlighting the need to better understand the current state of the literature and remaining knowledge gaps [5,6,9,10].

The long-term effects of the pandemic, coupled with ongoing cultural and academic stressors, necessitate further exploration. To the best of the investigative team’s knowledge, although previous systematic reviews have examined psychosocial adjustment among international students and compared mental health outcomes between domestic and international students [1,2], no previous scoping review has mapped the evidence with a sole focus on mental health outcomes among college-going international students in the United States since the start of the COVID-19 pandemic. A scoping review is particularly appropriate because the available literature encompasses diverse study designs, populations, and outcome measures, making evidence mapping more suitable than quantitative evidence synthesis. The purpose of the current project is to address this gap. We chose a scoping review over other types of reviews because it allows for a broad exploration of the literature without limiting the focus to particular study designs or interventions. This approach will help identify research gaps, key findings, and areas for future study.

Along those lines, the present review focuses on stress, anxiety, depression, and suicidal ideation because these outcomes are among the most prevalent and clinically significant indicators of psychological well-being in college populations and have been consistently examined among international students [1,2,20-23]. Stress reflects the immediate psychological demands associated with academic and cultural adjustment, whereas anxiety and depression are among the most prevalent mental disorders reported in college populations. Suicidal ideation represents one of the most serious adverse mental health outcomes and is an important public health concern because it may precede suicide attempts and death by suicide. Although other outcomes, including posttraumatic stress symptoms, sleep disturbances, substance use, and eating disorders, are also important, they have been investigated less consistently in studies of international students in the United States during the post-COVID-19 (2020) period, limiting opportunities for comprehensive synthesis.

### Objective

The objective of the current scoping review is to systematically map and characterize the available quantitative, qualitative, and mixed methods evidence on stress, anxiety, depression, and suicidal ideation among international college students enrolled in US colleges and universities during and following the COVID-19 pandemic. Specifically, the review will describe the extent, characteristics, and distribution of the available evidence. This includes summarizing study designs, participant characteristics, measurement instruments, and reported mental health outcomes, as well as identifying knowledge gaps to inform future research, practice, and policy.

## Methods

### Methodological Framework and Reporting Guidelines

This scoping review will follow the methodological framework outlined by Arksey and O’Malley [24], further refined by Levac, Colquhoun, and O’Brien [25]. These frameworks will guide each review stage, from identifying research questions to charting data and synthesizing findings. To ensure methodological rigor and transparency, this review protocol is registered on the Open Science Framework (OSF) [26] and is based on the Joanna Briggs Institute (JBI) guidelines for scoping reviews [27]. To promote clarity and completeness, the PRISMA-ScR (Preferred Reporting Items for Systematic Reviews and Meta-Analyses Extension Statement for Scoping Reviews; Checklist 1) will be used as the reporting guideline for the final scoping review manuscript [28]. In addition, the literature search strategy will be developed and reported in accordance with the PRISMA-S (Preferred Reporting Items for Systematic Reviews and Meta-Analyses Literature Search Extension) to promote transparent and reproducible reporting of the search methods.

### Eligibility Criteria

Studies will be eligible if they (1) include undergraduate or graduate international college students enrolled at US institutions of higher education; (2) examine one or more of the following mental health outcomes: stress, anxiety, depression, or suicidal ideation; (3) were conducted during the COVID-19 era (2020 onward); (4) use quantitative, qualitative, or mixed methods designs; and (5) are published as full-text, peer-reviewed articles in English. Studies published in or after 2020 that report data collected exclusively before the onset of the COVID-19 pandemic will be excluded. When studies include data collected across both prepandemic and pandemic periods, only findings relevant to the pandemic period will be charted when these are reported separately. Studies will be excluded if they focus exclusively on domestic students or populations outside the United States, were conducted before 2020, do not report one or more of the mental health outcomes of interest, are review articles, conference abstracts, case reports, gray literature, or are unavailable in full text.

### Rationale for Criteria

The focus on international students enrolled at US colleges and universities is intended to capture the unique academic, cultural, social, and immigration-related challenges experienced by this population within the US higher education system. Restricting studies to the COVID-19 era (2020 onward) ensures that the review reflects the pandemic and postpandemic context, during which international students encountered substantial disruptions to their educational experiences and mental well-being. The primary mental health outcomes of interest, namely stress, anxiety, depression, and suicidal ideation, were selected because they are among the most prevalent and clinically significant indicators of psychological well-being among college students, are closely associated with the acculturative and academic stressors experienced by international students, and have been consistently measured using validated instruments across diverse study designs, facilitating meaningful synthesis of the evidence. Including both quantitative and qualitative studies will allow for comprehensive mapping of the literature by capturing the prevalence of mental health outcomes alongside the lived experiences and contextual factors influencing international students’ well-being.

The search will be limited to full-text, peer-reviewed studies published in English-language journals. Previous methodological research has demonstrated that excluding non-English publications has minimal impact on the overall results and conclusions of systematic reviews [29,30]. Gray literature, including conference abstracts, dissertations, theses, and reports, will be excluded because these sources often provide limited methodological detail, have not undergone rigorous peer review, and contribute relatively few additional studies to evidence syntheses. Furthermore, previous methodological studies have found that excluding gray literature generally has little effect on the overall findings of reviews [31-33]. Restricting the review to peer-reviewed full-text publications is intended to enhance the methodological rigor, transparency, and reproducibility of the evidence synthesis. Although excluding gray literature may reduce the comprehensiveness of the evidence base, particularly for rapidly evolving topics such as COVID-19, restricting the review to peer-reviewed publications was considered appropriate to ensure methodological consistency, reproducibility, and the inclusion of studies that have undergone formal scientific review. The literature search will include studies published from January 2020 through December 2025.

### Search Strategy

A comprehensive literature search will be conducted in consultation with an experienced health sciences librarian. The search strategy will be developed using the population, concept, and context (PCC; Table 1) framework recommended by the JBI for scoping reviews and in accordance with the PRISMA-S to enhance transparency, reproducibility, and completeness of search reporting. Searches will be conducted by a health sciences librarian in the following electronic databases: PubMed (MEDLINE), PsycINFO, CINAHL Complete, Scopus, ERIC, and Web of Science Core Collection. The search strategy will combine controlled vocabulary (eg, MeSH in PubMed and subject headings in other databases, where applicable) with free-text keywords. Search terms will represent three primary concepts: (1) international college students, (2) mental health outcomes, and (3) COVID-19 within the context of higher education in the United States. Boolean operators (AND, OR), phrase searching using quotation marks, truncation (*), and database-specific syntax will be used as appropriate to maximize retrieval. The use of NOT as a Boolean operator will be avoided so as not to miss studies that may meet the inclusion criteria. Search strategies will be adapted to the indexing and search functionality of each database. The search will be limited to peer-reviewed, English-language studies published between January 1, 2020, and December 31, 2025. Conference abstracts, dissertations, theses, editorials, commentaries, letters, and other gray literature will be excluded. The reference lists of all included studies will be manually screened to identify additional eligible articles. No restrictions will be placed on study design. An example of a complete search strategy for PubMed is provided in Multimedia Appendix 1. Search strategies for the remaining databases will be adapted using equivalent controlled vocabulary and database-specific indexing terms. Search reporting will follow the PRISMA-S recommendations to enhance transparency and reproducibility.

**Table 1.** The population, concept, and context (PCC) framework was used to define the review scope and guide the development of the eligibility criteria and electronic search strategy.

PCC element	Example search terms
Population	International student, foreign student, overseas student, international graduate student, and international undergraduate student
Concept	Stress, anxiety, depression, suicidal ideation, and mental health
Context	COVID-19, SARS-CoV-2, pandemic, United States, college, and university

### Study Selection

Following the removal of duplicate records, titles and abstracts will be screened independently by 2 reviewers using predefined eligibility criteria. In Covidence, all retrieved citations will undergo title and abstract screening by 2 independent reviewers using the predefined eligibility criteria. Prior to formal screening, the reviewers will independently pilot-screen a random sample of approximately 50 citations to ensure consistent interpretation and application of the eligibility criteria. Any discrepancies identified during the pilot phase will be discussed, and the screening criteria will be refined as needed before full screening begins. Studies meeting initial eligibility will undergo full-text review by the same independent reviewers. Reasons for exclusion at the full-text stage will be documented and reported in the PRISMA (Preferred Reporting Items for Systematic Reviews and Meta-Analyses) 2020 flow diagram. Disagreements at either stage will be resolved through discussion, with consultation from a third reviewer when consensus cannot be reached.

### Roles of Collaborators

The review team consists of 1 principal investigator, 2 coinvestigators, and trained research assistants. Prior to screening, all reviewers will receive standardized training on the eligibility criteria and screening procedures. The principal investigator will oversee all stages of the review, including development of the search strategy, reviewer training, resolution of disagreements, and final approval of the extracted data and synthesized findings. Research assistants will participate in title and abstract screening and data charting under the investigators’ supervision.

### Data Charting

The standardized data charting form will be developed in Microsoft Excel and pilot-tested using 5 included studies. Revisions to the charting form will be documented before full data extraction begins. Any modifications made during the review process will be reported in the final manuscript. Studies reporting more than 1 eligible mental health outcome will contribute data to each applicable outcome category. All reported eligible outcomes (stress, anxiety, depression, and suicidal ideation) will be extracted independently to ensure comprehensive mapping of the available evidence.

### Data Synthesis

Extracted data will be synthesized using descriptive and narrative approaches consistent with the scoping review methodology. Descriptive summaries will report the frequency and distribution of studies according to publication year, study design, geographic location, sample characteristics, mental health outcomes assessed, and measurement instruments used. Narrative synthesis will be used to summarize reported findings related to stress, anxiety, depression, and suicidal ideation, as well as reported risk and protective factors. Evidence tables will summarize the characteristics of included studies, and graphical displays (eg, frequency charts or evidence maps) may be used to illustrate publication trends and the distribution of evidence across mental health outcomes. Findings will also be organized chronologically to describe how the literature has evolved from 2020 through 2025. Quantitative and qualitative findings will first be summarized separately. Quantitative studies will be synthesized descriptively using frequencies and summary tables, whereas qualitative findings will be synthesized narratively by identifying recurring themes related to stress, anxiety, depression, suicidal ideation, and contextual experiences. Findings from both evidence streams will then be integrated within the overall narrative synthesis to provide a comprehensive understanding of the mental health experiences of international college students. No formal qualitative synthesis methods (eg, meta-ethnography or thematic synthesis) will be conducted because the objective of this scoping review is to map rather than interpret or aggregate evidence.

### Risk of Bias Assessment

Consistent with the methodological guidance for scoping reviews provided by the JBI, a formal assessment of methodological quality or risk of bias will not be conducted. The primary purpose of this review is to systematically map the extent, range, and characteristics of the available evidence rather than evaluate the effectiveness of interventions or determine the certainty of the evidence. Consequently, studies will not be excluded based on methodological quality, and no critical appraisal tool will be applied. Additionally, the authors are not comfortable with their own characterization of studies based on the use of terms such as “low,” “moderate,” or “high” given the arbitrariness of such labels.

### Handling of Linked or Sibling Studies (Friend Studies)

In cases where 2 or more publications report findings from the same participant sample, cohort, or dataset (commonly referred to as friend, kin, or sibling studies), the review team will group these reports under a single primary study identifier to avoid duplicate data extraction. The primary publication will be selected based on the completeness of reporting (eg, sample characteristics, mental health outcomes, and methodological details). If multiple reports provide comparable information, the most recent publication will be used as the primary source. Relevant supplementary information from linked publications will be cross-referenced to ensure that no unique or relevant data are omitted. When separate reports from the same dataset present distinct, nonoverlapping findings related to different eligible mental health outcomes or participant characteristics, data from both reports will be extracted and charted together under the same study identifier rather than treated as independent studies. The relationship between linked publications, the rationale for selecting the primary publication, and any supplementary information extracted from related reports will be documented in the data charting form and described in the final scoping review to ensure transparency and reproducibility.

### Protocol Deviations

Any deviations from this protocol that occur during the review process will be documented, justified, and reported in the final manuscript.

### Dissemination

The findings of the completed scoping review will be disseminated through publication in a peer-reviewed journal and at least 1 presentation at a national or international scientific conference. The review findings may also be shared with higher education stakeholders, including university administrators, student affairs professionals, mental health practitioners, and policymakers, to support evidence-informed strategies aimed at improving mental health services for international college students. Finally, as previously noted, the protocol for this scoping review has been prospectively registered on OSF to promote transparency and facilitate access to the review methodology.

## Results

The a priori protocol for this unfunded scoping review was registered with the OSF. The review was conducted from December 25, 2025, through April 17, 2026. Following protocol registration, 2 additional reviewers were added to assist with study screening and data abstraction. Following screening, 253 records underwent further eligibility assessment, and 16 studies met the eligibility criteria for inclusion. Data analysis and synthesis have been completed, and the findings and discussion are currently being revised and formatted for submission of the completed scoping review. The completed review will present an evidence map describing study characteristics, reported mental health outcomes, methodological approaches, and knowledge gaps, with a focus on stress, anxiety, depression, and suicidal ideation among international college students enrolled in US institutions since the onset of the COVID-19 pandemic. Publication of the completed scoping review is tentatively anticipated by December 31, 2026.

## Discussion

### Principal Contribution

The current protocol describes a transparent and reproducible methodology for a scoping review that will systematically map the available evidence on stress, anxiety, depression, and suicidal ideation among international college students enrolled in US institutions during and following the COVID-19 pandemic. The completed review is expected to provide a comprehensive overview of the extent, characteristics, and distribution of the existing literature, including the study designs used, mental health outcomes assessed, measurement instruments used, and populations represented. By mapping the available evidence rather than evaluating intervention effectiveness, the review will identify areas where evidence is well established, highlight gaps in the literature, and inform priorities for future research, institutional practice, and policy development. This review is particularly timely given the increasing recognition of mental health as a critical component of international student success and well-being within higher education settings [3,4].

### Comparison With Previous Literature

Previous evidence syntheses have examined psychosocial adjustment among international students [2,19] and compared the mental health and well-being of domestic and international tertiary students [1]. These reviews have demonstrated that international students experience numerous academic, cultural, financial, and social challenges that influence psychological well-being. More recent studies conducted during the COVID-19 pandemic have further documented the effects of travel restrictions, visa uncertainty, social isolation, discrimination, and disruptions to academic experiences on international students’ mental health [9,10,20-23]. However, to our knowledge, no scoping review has specifically mapped the literature related to stress, anxiety, depression, and suicidal ideation among international college students enrolled in US institutions during and following the COVID-19 pandemic. By focusing on studies published between 2020 and 2025, this review will complement previous systematic reviews by describing the evolving evidence base, identifying methodological trends, and highlighting areas that may warrant additional investigation.

### Strengths and Limitations

The present protocol has several strengths. First, the review follows established methodological guidance from Arksey and O’Malley [24], Levac et al [25], and the JBI [27], thereby promoting methodological rigor and transparency throughout the review process. Second, prospective registration of the protocol in the OSF and planned reporting according to the PRISMA-ScR [28] and the PRISMA-S guidelines will enhance transparency, reproducibility, and completeness of reporting. Third, the comprehensive search strategy across 6 bibliographic databases, combined with both controlled vocabulary and free-text terms, is designed to maximize retrieval of relevant studies. Finally, the inclusion of quantitative, qualitative, and mixed methods research will enable a broad mapping of both the prevalence of mental health outcomes and the contextual experiences influencing international students’ well-being.

In addition to strengths, several potential limitations should also be acknowledged. First, restricting the review to English-language, peer-reviewed publications may exclude some relevant evidence. However, previous methodological studies have shown that excluding non-English publications generally has minimal impact on the overall conclusions of evidence syntheses [29,30]. Second, excluding conference abstracts, dissertations, and other gray literature will result in the exclusion of unpublished findings, although previous research suggests that these exclusions often have limited influence on review conclusions while improving methodological consistency and reproducibility [31-33]. Third, restricting the review to studies conducted in the United States during the COVID-19 era may also limit the generalizability of findings to other national contexts or earlier time periods. Finally, consistent with guidance for scoping reviews, no formal methodological quality or risk of bias assessment will be conducted, given that the purpose of a scoping review is to map the available evidence rather than evaluate the certainty of reported findings [27].

### Future Directions

The completed review will help identify understudied populations, methodological limitations, and inconsistently measured mental health outcomes that warrant further investigation. It will also inform future systematic reviews, meta-analyses, longitudinal studies, and intervention research focused on improving the mental health and well-being of international college students. Additionally, the evidence map generated through this review may assist higher education institutions, clinicians, and policymakers in identifying priority areas for culturally responsive mental health services, campus support programs, and institutional policies that address the unique challenges experienced by international students [4-6,8].

### Conclusions

This protocol outlines a transparent and reproducible methodology for systematically mapping the available evidence on stress, anxiety, depression, and suicidal ideation among international college students enrolled in US institutions during and following the COVID-19 pandemic. By following established scoping review methodology and reporting standards, including the JBI guidance and PRISMA-ScR recommendations, the completed review will provide a comprehensive evidence map of the existing literature and identify methodological trends and knowledge gaps that warrant further investigation [24,25,27,28].

The resulting evidence synthesis has the potential to inform future research, guide the development of systematic reviews and intervention studies, and support evidence-informed decision-making within higher education. In addition, findings from the completed review may assist university administrators, student affairs professionals, mental health practitioners, and policymakers in developing culturally responsive services and institutional strategies that address the unique mental health needs of international college students [3-6,8]. Overall, this scoping review will contribute to a clearer understanding of the current state of the literature and provide a foundation for advancing research, practice, and policy aimed at promoting the mental health and well-being of international college students in the United States.

## Supplementary material

10.2196/91839 Multimedia Appendix 1 PubMed search strategy.

10.2196/91839 Checklist 1 PRISMA-ScR checklist.

## Abbreviations

JBI: Joanna Briggs Institute
OSF: Open Science Framework
PCC: population, concept, and context
PRISMA: Preferred Reporting Items for Systematic Reviews and Meta-Analyses
PRISMA-S: Preferred Reporting Items for Systematic Reviews and Meta-Analyses Literature Search Extension
PRISMA-ScR: Preferred Reporting Items for Systematic Reviews and Meta-Analyses Extension for Scoping Reviews
