# Supplementary material for: Mental Health Outcomes Among International College Students in the COVID-19 Pandemic: Protocol for a Scoping Review
Source: JMIR Res Protoc. 2026 Sep 25;15:e91839. doi: 10.2196/91839 (PMC13614222; doi:10.2196/91839)
Supplement: Supplementary file 2 [file resprot-v15-e91839-s001.docx]

**Multimedia Appendix 1: PubMed Search Strategy**

(

("Students"[Mesh] OR student* OR undergraduate* OR graduate student* OR college student* OR university student*)

AND

("International Educational Exchange"[Mesh] OR international student* OR foreign student* OR overseas student*)

)

AND

(

"Mental Health"[Mesh]

OR "Depression"[Mesh]

OR "Anxiety"[Mesh]

OR "Stress, Psychological"[Mesh]

OR "Suicidal Ideation"[Mesh]

OR mental health

OR stress

OR anxiety

OR depression

OR depressive symptoms

OR suicidal ideation

)

AND

(

COVID-19

OR SARS-CoV-2

OR coronavirus

OR pandemic

)

AND

(

United States

OR US

OR USA

OR American college*

OR American universit*

)

**Filters applied:** English, Journal Article

Publication Date: 2020/01/01 to 2025/12/31

Humans
